# Canine Morphology: Hunting for Genes and Tracking Mutations

**DOI:** 10.1371/journal.pbio.1000310

**Published:** 2010-03-02

**Authors:** Abigail L. Shearin, Elaine A. Ostrander

**Affiliations:** 1 National Human Genome Research Institute, National Institutes of Health, Bethesda, Maryland, United States of America; 2 University of Pennsylvania School of Veterinary Medicine, Philadelphia, Pennsylvania, United States of America

## Abstract

In this essay, Abigail Shearin and Elaine Ostrander discuss the proposed genomic mechanisms for the extraordinary level of phenotypic variation observed in the domestic dog and the evidence detailing the variants responsible for the many shapes, sizes, textures, and colors of man's best friend.

## Summary

As a result of domestication, selection for desirable phenotypes, and breed propagation, the domestic dog is unmatched in its diversity as a land mammal. Exhibiting extraordinary levels of both interbreed heterogeneity and intrabreed homogeneity, evidenced in part by the extensive linkage disequilibrium observed in many breeds, the dog provides an as-yet unrealized opportunity to uncover the molecular mechanisms that govern natural variation across mammalian species. We herein discuss recent advances in canine genomics that have made exploration of genetic mechanisms controlling breed-specific differences possible. We consider some examples where molecular mechanisms controlling simple traits have been uncovered. Finally, we reveal how combinations of genes produce complex phenotypes that can be revealed through studies of dog breeds featuring specific traits.

## Introduction

**Figure 1:**
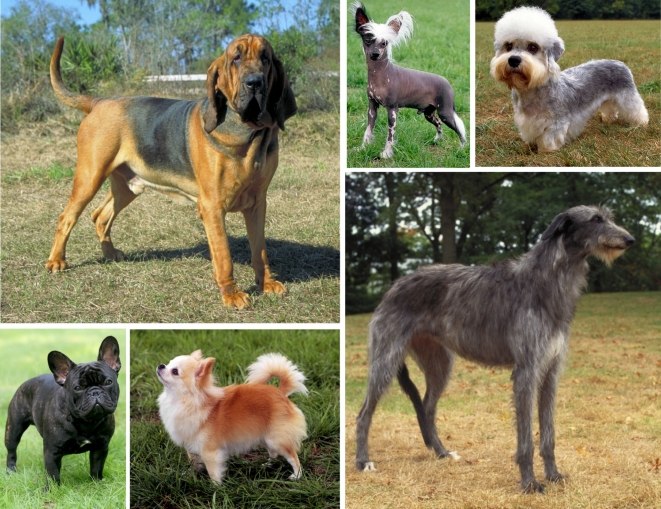
Morphological variation in the dog. Dog breeds display extremes of morphological variation including body size and proportion, head size and shape, coat texture, color, and patterning. Clockwise from the left: the Bloodhound, the Chinese-crested, the Dandie Dinmont terrier, the Scottish deerhound, the long-haired Chihuahua, and the French bulldog. (Image: Mary Bloom, American Kennel Club).

As Darwin himself noted, the domestic dog displays a remarkable level of phenotypic diversity [1], and it is arguably the most morphologically variable land mammal on the earth today. Dogs can be big or small, tall or short, and display extremes of variation in terms of coat color and texture, skull shape and size, leg length and width, and a host of other traits (Figure 1). How this variation developed and is maintained within breeds intrigues both scientists [2]–[5] and the lay public alike.

There are over 300 dog breeds identified worldwide, with nearly 170 recognized in the United States by the American Kennel Club (AKC) [6]. All domestic dog breeds are members of the same species, *Canis familiaris*, and possess a 2.8 Gb genome featuring 38 autosomes and the sex chromosomes, similar in size to the 3 Gb human genome. Dogs of any breed can, for the most part, be crossed to produce fertile offspring. Breeds were developed largely during the Victorian era, with special selection for both morphologic traits based on size, proportion, coat, etc., as well as behavior. To be a registered member of a breed, both of a dog's parents have to be registered members of the same breed, and their parents in turn must be registered members of the breed. Thus, each breed is effectively a closed breeding population that offers many statistical advantages for doing genetics beyond what can be done in studies of human populations [7].

In this essay we consider some of the features of the canine genome relevant for successful studies of selected traits. We discuss current hypotheses regarding the development and maintenance of genetic variation in dogs today. We consider examples in which identified genes account for unique, and sometimes complex, phenotypes. Finally, we consider the implications of these findings for studies of true complex traits, such as those associated with behavioral genetics.

## The Canine Genome and Linkage Disequilibrium

The canine genome was sequenced to both 2× [8] and 7.8× density [9] in the standard poodle and boxer, respectively. The average nucleotide heterozygosity, when considered across dog breeds, is 8×10^−4^, which is essentially the same high level of nucleotide diversity reported in the human population. As expected, however, the level of genetic diversity within any single breed is considerably less than the species as a whole [10]. Most breeds demonstrate a pattern indicative of two population bottlenecks—domestication and breed formation [9],[11]. In support of that, Gray et al. modeled the demographic history of wild canid populations and domestic dog breeds and showed that domestication resulted in a 5% loss of nucleotide diversity, while breed formation caused a 35% loss [11].

**Figure 2:**
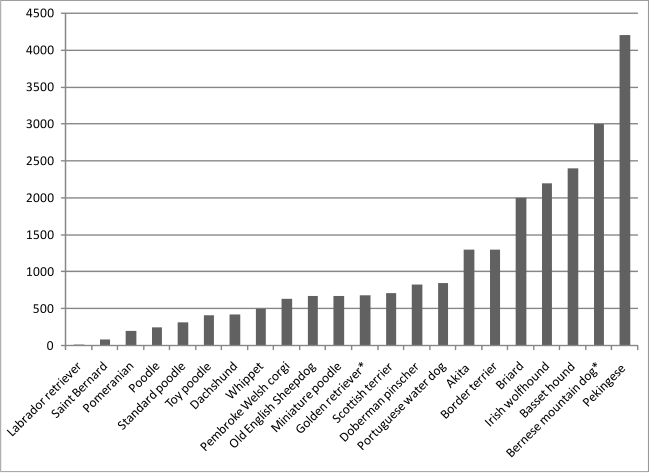
Linkage disequilibrium in the dog. The average LD distances were established by Gray et al. for several breeds based on the distance, where r^2^ decays to two [11]. LD distances for breeds denoted with an (*) were established previously by Sutter et al. and are based on the distance where D′ falls to half its maximum value [12]. The degree to which LD varies between breeds is remarkable and the fine mapping of traits is greatly facilitated when data from multiple breeds can be combined. The level of LD within a breed can be attributed to a number of factors: the historical use and popularity of the breed; the effective population size; bottlenecks due to size of the starting population; popular sire effects; and breeding practices which allow matings between closely related individuals.

The loss of diversity reported by Gray et al. [11] is evident in the extensive linkage disequilibrium (LD), the nonrandom association of alleles at two or more loci, that is reported among breeds [9],[12] (Figure 2). LD within a single dog breed can extend for megabases (Mb), compared to the 20–50 kb that is more typically observed in humans [9],[12],[13]. Not surprisingly, extensive haplotype sharing between breeds is also observed, as many breeds derive from combinations of breeds, followed by strong selection for specific phenotypes.

The extensive LD that characterizes dogs means that genome-wide association studies (GWAS) can be done in the dog with as few as 20,000–30,000 single nucleotide polymorphisms (SNPs), compared to the million needed for the more outbred human population [9],[12]. Once a locus is identified, it takes a significant amount of luck to find the causative mutation if only one breed is considered. The analysis of multiple breeds that share a common ancestral mutation, however, can quickly reduce a region from Mb to kb [10],[13]–[16]. These facts, coupled with the small number of samples required for a canine GWAS [9] means that identification of variants underlying complex morphologic phenotypes can be accomplished in the dog with a fraction of the investment typically required for a comparable human study.

To aid in the selection of breeds for any given study we recently did a cluster analysis of 132 dog breeds and showed that breeds divide into five major groups: Asian and ancient dogs; hunting and gun; mastiff and terrier; herding and sight hound; and a mountain group [10]. Dogs from the same cluster often carry the same ancient mutation. Thus, judicious selection of breeds for fine-mapping studies can greatly reduce both work load and complexity associated with the study [10].

## The Dog Genome and Phenotypic Variation

### Traits of the Canine Genome

The closest relative to the domestic dog is undeniably the gray wolf, from which the dog differs by only 0.04% in nuclear coding-DNA sequence [9],[17],[18]. Phylogenetic studies of mtDNA from domestic dogs and wolves demonstrate multiple backcrossing events that occurred 15,000–100,000 years ago [18],[19]. Given that, one can ask if the gray wolf of today contains all the diversity needed to create the variation observed in modern domestic dogs. Alternatively, is there something intrinsically unique about the canine genome such that it generates a rapid rate of non-lethal mutations associated with unique phenotypes for breeders to select? Artificial selection is common among all domestic animals but no others exhibit the level of variation observed in the dog. Dogs were domesticated earlier than probably any other species. However, artificial selection for breed standards began during the Victorian era, suggesting that astute breeders, new mutations, and strong selection based on breed standards over the past 200 years have all contributed to the morphological variation observed in dogs today. The vast array of traits exhibited across dog breeds makes a compelling argument that some innate mechanism for variation was present prior to the intense selection over the past 200 years, whether unique to the dog or also present in the wolf genome.

The degree to which new mutations have played a role in the development of the modern dog still requires intense scrutiny, but three major sources of genomic variation have been proposed as contributors to the high levels of phenotypic variation observed in today's domestic dogs. The first is variability associated with microsatellites or simple sequence repeats (SSRs). Fondon and Gardner hypothesized that repeat length polymorphisms, particularly those occurring in regulatory regions, were an important source of morphologic variation, in part because they occur at a mutation rate 100,000× greater than SNPs [20]. Their contention is supported by a comparison of repeat lengths between humans and dogs at 36 developmentally associated loci, which revealed significant recent changes in the length of the dog alleles. When these same loci were compared amongst dog breeds, five genes exhibited large repeat expansions or contractions. Among the more interesting was a polymorphism observed in Great Pyrenees within the coding sequence of the *Alx-4* gene, which is postulated to be responsible for their characteristic rear digit polydactyly [20]. Four Great Pyrenees exhibiting rear digit polydactyly possessed the variant *Alx-4* allele, while one Great Pyrenees who lacked rear digit polydactyly did not carry the variant allele [20].

Elaborating on this theme, the same investigators found that members of the Canidae family possessed elevated genome-wide basal slippage rates, the rate at which DNA replication machinery creates new alleles due to errors in replicating repeat elements, compared to humans, non-human primates, and other members of the Carnivora order (i.e., cats) [21]. In addition, several Canidae-specific slippage events were clade-specific. Repeat sequences in wild canids were nearly identical to those observed in the dog, whereas more distantly related members of the Carnivora order displayed less repeat purity. For example, in the Felidae family, repeat purity was approximately 93%, versus a Canidae average repeat purity of 95%. The authors suggest that this is indicative of an accelerated loss of ancestral repeat impurities in the Canidae lineage. This in turn suggests that mutations accumulated as a result of repeated basal slippage events occurring over millions of years, and not as a result of a rapid rise of the basal slippage rate [21]. Thus, the amount of phenotypic variation observed in the domestic dog may be attributable to particular features ubiquitous to the wolf genome.

Another mechanism that clearly accounts for a subset of diversity between breeds is carnivore-specific short-interspersed nuclear elements (SINEs) [22]. In the dog, the most common such element, denoted SINEC_Cf, makes up 7% of the total genome sequence [8],[23]. Whereas the total percentage of SINEs in the canine genome is slightly less than in the feline genome [24], the activity levels of the felid-specific SINEs FC1 and FC2 versus the SINEC_Cf in the dog have not been explored. Rodent SINEs such as B1 in the mouse and rat genomes have been found to have a high level of activity [25]. However, these SINEs comprise a smaller percentage of the total genome than the comparable sequences in the dog, with only 1.5% of the mouse genome being comprised of lineage specific SINEs [25]. The abundance of SINEC_Cf in the canine genome, coupled with its characteristic low divergence, indicates a likely recent expansion event [8],[23] and makes SINEC_Cf elements an excellent candidate for genetic diversity in the dog. This is supported by the work of Lindblad-Toh et al. who showed that 10,000 SINE insertion sites are bimorphic between the boxer and standard poodle [9]. Further support comes from empirical data. SINE insertions have been shown to be responsible for several canine diseases, including narcolepsy [26] and centronuclear myopathy [27], as well as some morphologic features such as merle coat color [28] and possibly white spotting on the coat [13]. A study of the abundance and level of activity of SINEs in the wolf genome would provide additional insight. These three proposed mechanisms of canine variation demonstrate that the dog genome is unique from other mammalian genomes and may be unique in its rate or process for producing new mutations.

### Common Sources of Variation

Other possible mechanisms of variation in the dog are common to many species and include mutational hotspots, chromosomal fission, and gene duplications. The latter are particularly interesting. For instance, duplication of a 133-Kb region spanning three fibroblast growth factor genes was shown to be associated with the appearance of a characteristic ridge on the back of the Rhodesian ridgeback breed [29]. Another interesting example is the expression of a *fibroblast growth factor-4* (*fgf4*) retrogene, a gene copied by reverse transcriptase from processed mRNA and inserted into the genome, which we demonstrated is associated with chondrodysplastic breeds displaying disproportionately short limbs [30]. The trait appears fixed in nearly 20 breeds including the corgi, dachshund, Scottish terrier, and basset hound. To identify the underlying variant, we performed a large GWAS [30]. Analysis of the data revealed a locus on canine Chromosome 18 (CFA18) that spanned several genes, none of which were particularly provocative. Haplotype analysis following additional SNP genotyping reduced the critical region to 24 Kb, with evidence of a selective sweep, a reduction in genetic variation in the region surrounding a gene under strong selection. Sequencing of the region revealed a retrogene that contained the complete coding sequence of *FGF4* but none of the introns or regulatory machinery. Expression studies revealed that the adjacent genes were expressed in neonatal chondrocytes, as was the retrogene. However, the retrogene was not expressed in the cartilage of mature dogs. Although expressed retrogenes are common in insects, this is the first example we are aware of in which alleles of a retrogene segregate in a mammalian species such that they are a major source of morphological variation [30].

### Skeletal Morphology

The first large-scale genetic studies of canine skeletal morphology were done by Chase et al., in the Portuguese Water Dog (PWD) [31]. They sampled over 500 PWDs from whom they collected a set of 92 X-ray–based metrics. They then did a genome wide scan, which when aligned with a principal components analysis (PCA) identified quantitative trait loci (QTLs) for several complex traits including body size, leg length versus width, and skull shape.

**Figure 3:**
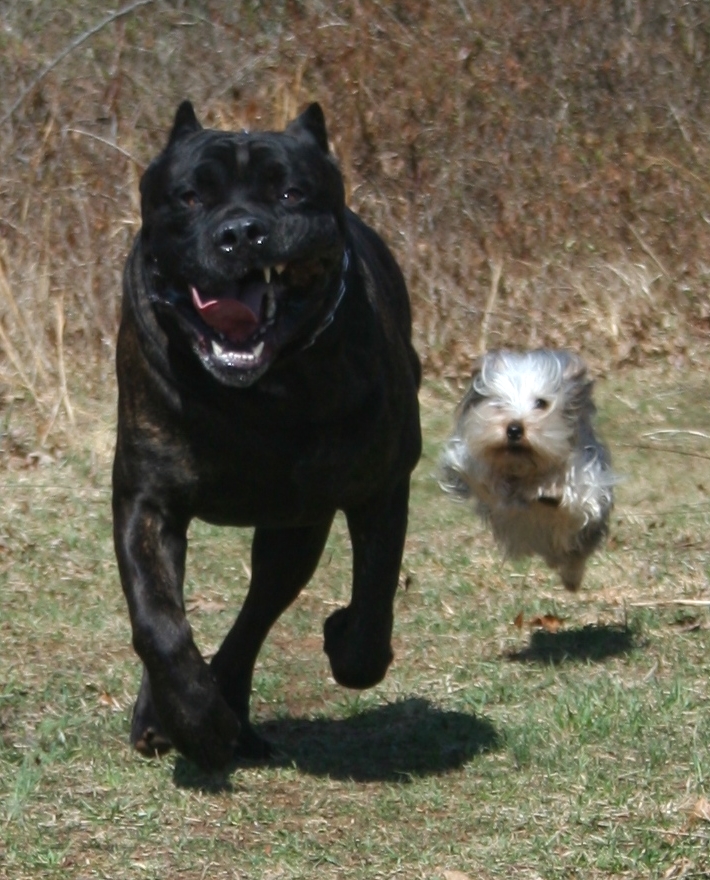
Size variation in the dog. Variation in skeletal morphology in the dog is a complex phenotype, with *IGF-1* as a major determinant of small size [32]. The difference in overall body size between a Cane Corso and a Yorkshire terrier is over 30-fold, yet both are members of the same species, *Canis familiaris*.

We showed that the primary signal for body size (PC1) was a four million base pair (bp) locus spanning several genes on CFA15 [31],[32]. Additional genotyping of large and small PWD narrowed the region, and analysis of size-selected breeds (large versus small), revealed a selective sweep in 14 small breeds (i.e., Pekingese, toy poodle, etc.) that precisely spanned the *insulin like growth factor-1* (*IGF-1*) gene [32] (Figure 3). A single *IGF-1* haplotype was shared among all small dogs, suggesting that a single ancestral mutation had been selected for in the development of all small dog breeds studied. Two distinct *IGF-1* haplotypes segregated in large dogs, suggesting a more complicated scenario for enlarging breed size [32]. At least four additional loci contribute to overall skeletal size in the dog and are currently under analysis [31],[33].

Additional skeletal traits studied include PC2, which defines leg length versus width in the original study of Chase et al., [31] and which maps to a locus on CFA12 [34]. Additional studies in both PWDs and size-selected breeds representing phenotypic extremes of PC2 reduced the region from 26 Mb to 500 kb [34]. The proximity of the critical interval to two collagen genes suggests that the phenotype may be controlled by *cis*-acting mechanisms, although the critical mutation remains to be found.

### Fur Texture and Color

**Figure 4:**
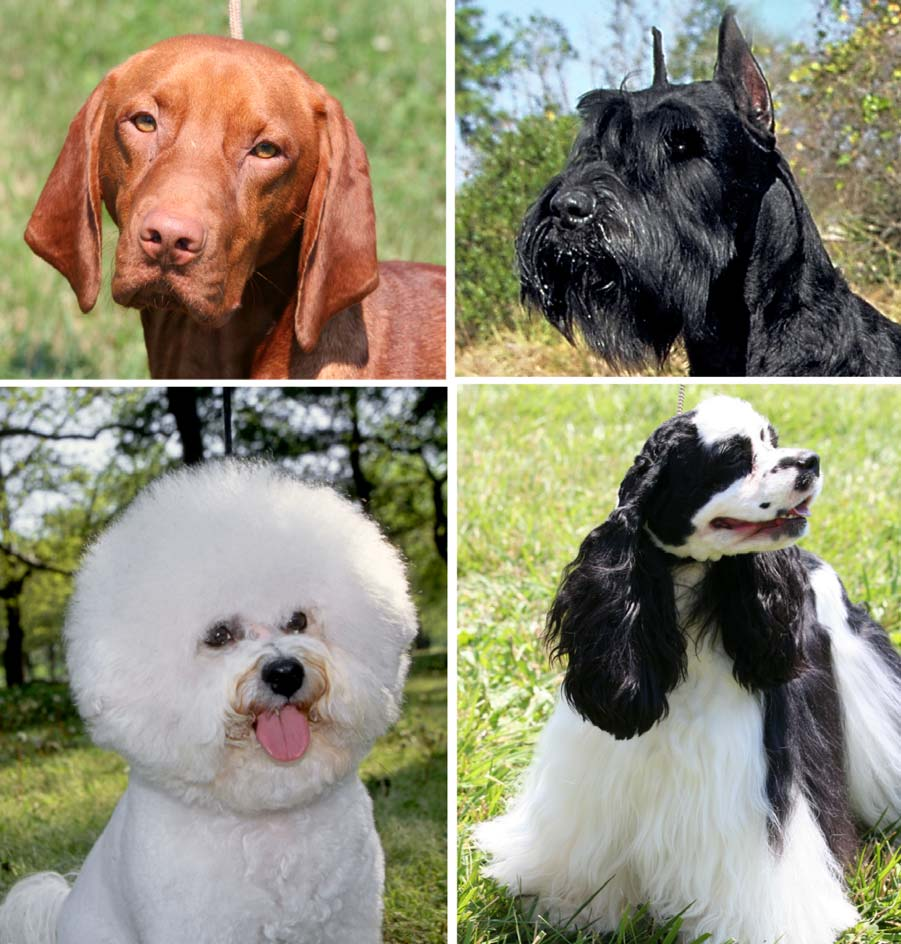
Coat variation in the dog. Coat texture and length are features that distinguish between breeds and between varieties of the same breed [16]. Clockwise from the left are shown the Vizsla with a short, straight coat. These dogs and others like them have wild-type alleles for the three critical genes controlling coat texture, length, and curl, which are *RSPO-2*, *FGF5*, and *KRT71*, respectively. The giant Schnauzer displays the eyebrows and moustache characteristic of the trait called “furnishings” and carries the variant form of *RSPO-2*. Dogs with furnishings usually exhibit wiry coats as well. The Cocker spaniel has long straight hair, demonstrating the variant form of *FGF5*, but wild-type alleles at other loci. The Bichon frise has variant alleles at all three critical loci, *RSPO-2*, *FGF5*, and *KRT71*, and displays a coat that is long, curly, and with furnishings. (Image: Giant schnauzer and Bichon frise pictures provided by Mary Bloom, American Kennel Club.)

Some of the most exciting progress in understanding the genetics of variation in dogs relates to the complex traits of coat texture and color. We recently showed that variation in canine pelage, including pattern, length, curl, and texture (smooth versus wire), are controlled by combinations of alleles at only three genes [16] (Figure 4). A 167-bp deletion at the 3′ end of the *R*-*spondin-2* (*RSPO2*) gene is strongly associated with wire hair and “furnishings”, the latter being the moustache and eyebrows characteristically seen, for instance, in the schnauzer [16] (Figure 4). Long versus short fur is associated with a (Cys95Phe) change in exon one of the *fibroblast growth factor-5* (*FGF5*) gene. Curly versus straight fur is associated with a coding SNP within the *keratin71* (*KRT71*) gene [16], as it is in mice [35].

Remarkably, combinations of alleles at just these three genes account for ∼95% of coat variation observed among the 108 AKC breeds studied. For instance, the Bichon frise carries the variant allele for *RSPO2*, *FGF5*, and *KRT71* and thus possesses long, curly hair with furnishings (Figure 4). One additional source of variation, a lack of coat as seen in the Chinese crested, Mexican and Peruvian hairless breeds is explained by a frame shift mutation in *FOX13*, a member of the fork head box transcription factor family [36].

Coat color is independent of type and is primarily governed by the melanocortin 1 receptor (Mc1r) pathway. Variants result from mutations in the *Agouti*, *Mc1r*, and *CBD103* genes, the latter of which encodes β-defensin. A coat like that of the German shepherd contains both black and yellow pigments, termed eumelanin, and pheomelanin, respectively. Coats expressing only pheomelanin develop when Mc1r is nonfunctional and therefore unable to produce eumelanin [37],[38]. Coats expressing only eumelanin occur via two mechanisms: recessive black coats are observed when the agouti protein is nonfunctional. Dominant black coats occur when a derived β-defensin protein competitively inhibits the agouti protein [39],[40].

Several dog breeds exhibit complete or partial absence of pigmentation. For instance, Karlsson et al. mapped a locus for white-spotting to a 102-kb haplotype on CFA 20 in a region that spans a single gene; *microphthalmia-associated transcription factor* (*MITF*), which is crucial for melanocyte migration [13]. Two potential mutations were identified, one of which is a SINE insertion that may disrupt transcription [13].

## Conclusions

The identification of genetic variants controlling morphology in the dog population has reached an exciting juncture. The current set of available molecular tools allows us to finally address the critical questions. For instance, the striking morphological variation observed between breeds of dogs provides us with unique opportunities to study the genetic basis of both evolution and domestication. A deeper understanding of the genomics and variation in wild canids would enhance our ability to pursue these questions.

Several hypotheses have been proposed as to why the dog, as opposed to any other domestic land mammal or any other domesticated creature, displays such extremes of morphologic variation (Box 1). Each theory has its champions, and most likely a combination of mechanisms contribute with the strong artificial selection imposed by man being the most important. The question remains: if under the same intense artificial selection for novel morphological traits, would other domestic creatures exhibit equivalent variation? As scientists continue hunting for the genes and tracking the mutations that control morphologic variation in the domestic dog, we expect still more secrets will be revealed regarding the genetic basis of man's extraordinary best friend.

**Box 1. Possible Mechanisms of Canine Variation**

- Microsatellites or simple sequence repeats
- High levels of repeat purity
- The abundance and location of SINEC_Cf elements in the canine genome
- Common sources of variation: mutational hotspots, chromosomal fissions, and gene duplications
- Intense artificial selection
- Rapid perpetuation of new mutations

## Abbreviations

AKC: American Kennel Club
GWAS: genome-wide association study
FGF4: fibroblast growth factor-4
FGF5: fibroblast growth factor-5
IGF-1: insulin like growth factor-1
KRT71: keratin71
LD: linkage disequilibrium
Mc1r: melanocortin 1 receptor
PWD: Portuguese Water Dog
RSPO2: R-spondin-2
SINE: short-interspersed nuclear element
SNP: single nucleotide polymorphism
SSR: simple sequence repeat
